# Characterization and engineering of a dual-function diacylglycerol acyltransferase in the oleaginous marine diatom *Phaeodactylum tricornutum*

**DOI:** 10.1186/s13068-018-1029-8

**Published:** 2018-02-09

**Authors:** Yulin Cui, Jialin Zhao, Yinchu Wang, Song Qin, Yandu Lu

**Affiliations:** 1 Key Laboratory of Coastal Biology and Biological Resource Utilization, Yantai Institute of Coastal Zone Research, Chinese Academy of Sciences, Yantai, 264003 Shandong China; 2 State Key Laboratory of Marine Resource Utilization in South China Sea, College of Oceanology, Hainan University, Haikou, Hainan 570228 China; 3 University of Chinese Academy of Sciences, Beijing, 101408 China

**Keywords:** *Phaeodactylum tricornutum*, WS/DGAT, TAG, Wax ester, Metabolic engineering

## Abstract

**Background:**

Photosynthetic oleaginous microalgae are promising feedstocks for biofuels. Acyl-CoA:diacylglycerol acyltransferases (DGATs) represent rich sources for engineering microalgal lipid production. The principal activity of DGATs has been defined as a single-function enzyme catalyzing the esterification of diacylglycerol with acyl-CoA.

**Results:**

A dual-function PtWS/DGAT associated with diatom *Phaeodactylum tricornutum* is discovered in the current study. Distinctive to documented microalgal DGAT types, PtWS/DGAT exhibits activities of both a wax ester synthase (WS) and a DGAT. WS/DGATs are broadly distributed in microalgae, with different topology and phylogeny from those of DGAT1s, DGAT2s, and DGAT3s. In vitro and in vivo assays revealed that PtWS/DGAT, functioning as either a WS or a DGAT, exhibited a preference on saturated FA substrate. Endogenous overexpression of PtWS/DGAT demonstrated that the DGAT activity was dominant, whereas the WS activity was condition dependent and relatively minor. Compared with the wild type (WT), overexpression of PtWS/DGAT in the diatom resulted in increased levels of total lipids (TL) and triacylglycerol (TAG) regardless of nitrogen availability. The stability and scalability of the introduced traits were further investigated at a 10-L photobioreactor, where the mutant growth resembled WT, with moderately increased productivity of TL and TAG. Furthermore, the production of wax esters increased considerably (from undetectable levels to 2.83%) under nitrogen-deplete conditions.

**Conclusions:**

PtWS/DGAT is a bifunctional enzyme and may serve as a promising target for the engineering of microalga-based oils and waxes for future industrial use.

**Electronic supplementary material:**

The online version of this article (10.1186/s13068-018-1029-8) contains supplementary material, which is available to authorized users.

## Background

Microalgae sequester the carbon dioxide in the atmosphere, converting this gas into green chemicals for scalable production of valuable molecules ranging from therapeutic proteins to biofuels [1]. Triacylglycerol (TAG) is an energy-dense lipid and a promising source for biofuel production from microalgae [2]. However, few natural strains exhibit the demanding traits associated with feedstock for TAG accumulation and, hence, genomic and biological technologies with increased specificity for microalga-based biofuel studies have been sought [3]. Identification of the pathways and crucial enzymes that underlie the oleaginous phenotype should guide the rational genetic engineering of microalgae for the overproduction of TAG [4, 5].

The unicellular microalga *Chlamydomonas reinhardtii* [6, 7], *Phaeodactylum tricornutum* [8–10], and *Nannochloropsis* sp. [11–14] have served as model species for investigating the genetic mechanism of TAG production. Acyl-CoA:diacylglycerol acyltransferases (DGATs) catalyze the final and committed step in TAG biosynthesis. The function of DGATs, mainly DGAT1s and DGAT2s, has been characterized in various studies [8, 9, 12, 14–29]. Despite functional differentiation, DGATs have been defined as single-function enzymes catalyzing the esterification of diacylglycerol (DAG) with acyl-CoA [12, 30].

Similar to TAG, in a number of plants and microorganisms, wax esters serve as energy reservoir which has been regarded as potential alternative energy sources for biofuel production. A dual-function enzyme wax ester synthase/acyl coenzyme A (acyl-CoA):diacylglycerol acyltransferase (WS/DGAT) has been discovered in some plants and bacteria [31–33], catalyzing the final step in TAG and wax esters biosynthesis. However, WS/DGAT enzyme has been very rarely reported so far in eukaryotic microorganisms [33] and has not been discovered in microalgae. The oleaginous microalga *P. tricornutum* harbors a single DGAT1 and four putative DGAT2s, of which DGAT1 [8] and DGAT2B [9] have been functionally characterized in vitro. This characterization revealed a distinct substrate preference with PtDGAT1 exhibiting a preference toward saturated C_16_ and C_18_ fatty acids (FAs) [8] and PtDGAT2B exhibiting specificity on unsaturated C_16_ and C_18_ FAs [9, 10]. In *P. tricornutum*, we discovered a group of acyl-CoA:diacylglycerol acyltransferases (designated as PtDGATX) with an identity high to WS/DGAT and low to DGAT1s, DGAT2s, and DGAT3s, in amino acid sequence. This suggests that the function of DGATXs differs from those of the remaining types of DGATs. However, identification of the molecular and cellular mechanisms of PtDGATX underlying TAG or wax ester metabolism remains challenging. Therefore, the aim of this study is to investigate PtDGATX function, substrate preference, and regulatory mechanisms in lipid biosynthesis. Distinctive substrate preferences and in-concert choreography in TAG or wax ester biosynthesis of individual DGATs are revealed. Furthermore, a genetically modified diatom with elevated lipid, wax ester, and TAG productivity is created. In particular, the engineered strains yield (although low) wax esters, a class of oleochemicals that can potentially be used for many applications in various industries [34, 35].

## Results and discussion

### DGATX distribution in microalgae

**Fig. 1:**
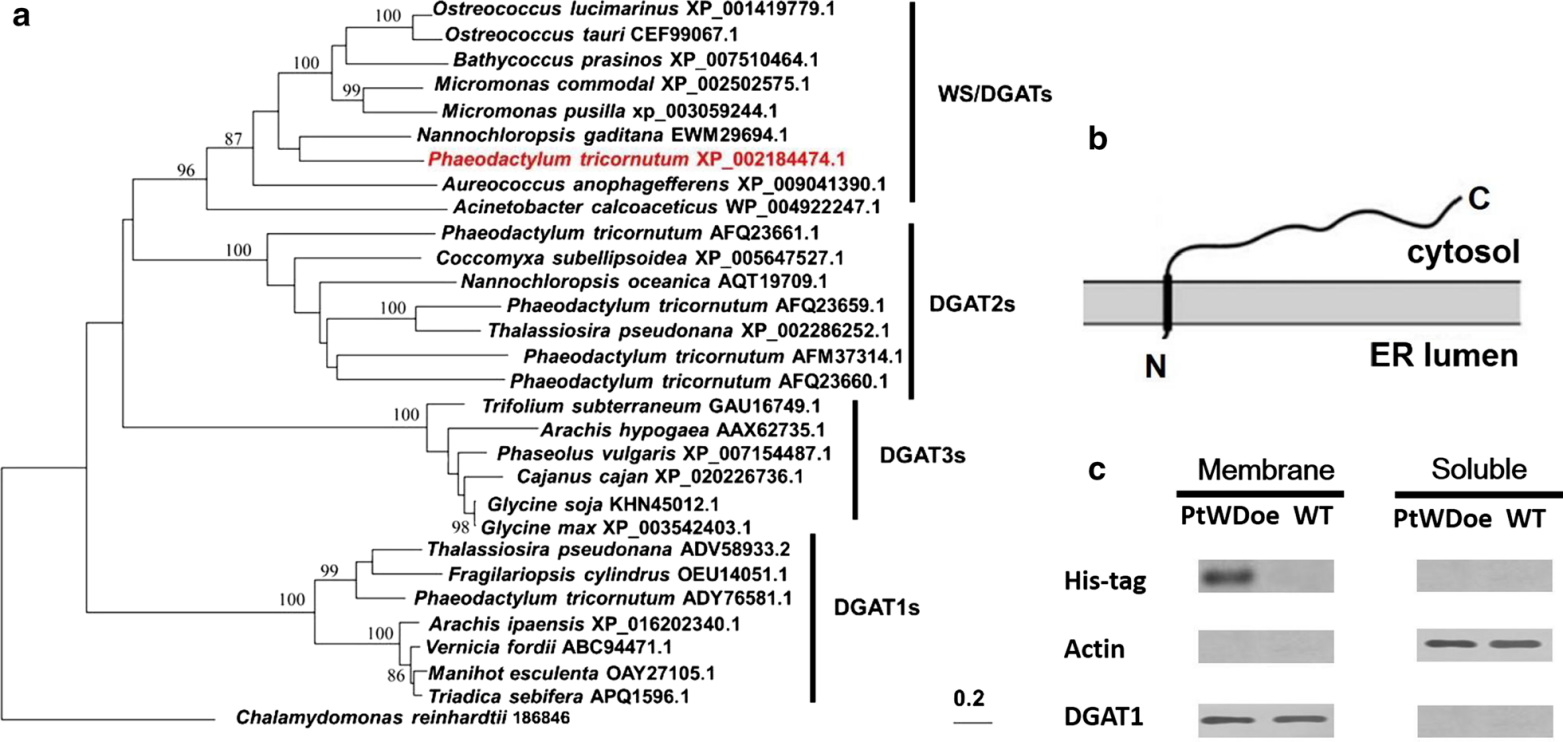
Sequence analysis of PtDGATX. **a** Phylogenetic analysis of DGATs. The PDAT gene of *Chlamydomonas reinhardtii* (186846) was used as the root at the outer group. Sequence alignment and phylogenetic analysis were performed with BLAST and CLUSTALW, respectively. Bootstrap support values were estimated using 100 pseudo-replicates. **b** The putative transmembrane construct of PtDGATX. The transmembrane region was analyzed by the online software TMHMM, DAS, and TMpred. **c** Topology analysis of PtDGATX via western blotting. Soluble and membrane proteins were separated and used for blotting. Actin and DGAT1 which were known soluble protein and membrane protein, respectively, were used as controls. *N* the N terminal, *C* the C terminal, *Membrane* transmembrane proteins, *Soluble* soluble proteins, *PtWDoe* the PtDGATX overexpression line of *P. tricornutum*, *WT* the wild-type *P. tricornutum*

The PtDGATX was characterized by first validating its gene model through a polymerase chain reaction (PCR) from cDNA and genomic DNA (Additional file 1: Dataset S1). The lack of intron suggested that the gene structure of PtDGATX was compact. The PtDGATX was distributed separately from (Fig. 1a) and was modestly similar to characterized microalgal DGAT1s, DGAT2s, and DGAT3s (Fig. 1a). This suggested that the origin and function of PtDGATX were distinct from those of DGAT1s, DGAT2s, and DGAT3s. The phylogeny of DGATXs revealed a broad presence and a high level of sequence conservation ranging from bacteria, microalgae to flowering plants (Additional file 2: Figure S1). A relatively high identity in amino acid sequence was revealed between DGATX and WS/DGAT (20%; bacterium *Acinetobacter,* AF529086; Additional file 2: Figure S2), which has been characterized in bacteria as a bifunctional enzyme WS/DGAT [32]. Moreover, the TIGR02946 domain of WS/DGATs [31] was present in PtDGATX. DGAT1s have at least six transmembrane domains [8] and are larger than DGAT2s, which have (in general) at least two putative transmembrane domains [36], while DGAT3s are cytosolic diacylglycerol acyltransferase in the absence of transmembrane domains [37]. PtDGATX resembled AcWS/DGAT, and was predicated to harbor a single hydrophobic transmembrane region in the N terminal (Val3-Met35) (Fig. 1b and Additional file 2: Figure S3a). This was validated by fusing the coding sequence of PtDGATX with a His tag. The fusion protein was expressed endogenously in *P. tricornutum* and the His-tag antibody was used to determine the localization of the PtDGATX protein via western blotting. Soluble and membrane proteins were separated and used for this blotting. Bands were absent from the soluble proteins, but a band occurred in the membrane proteins (Figs. 1c and Additional file 2: Figure S3b), suggesting that PtDGATX was a transmembrane enzyme. Altogether, in terms of topology and phylogeny, PtDGATX is more similar to WS/DGATs than to DGAT1s, DGAT2s, and DGAT3s.

**Fig. 2:**
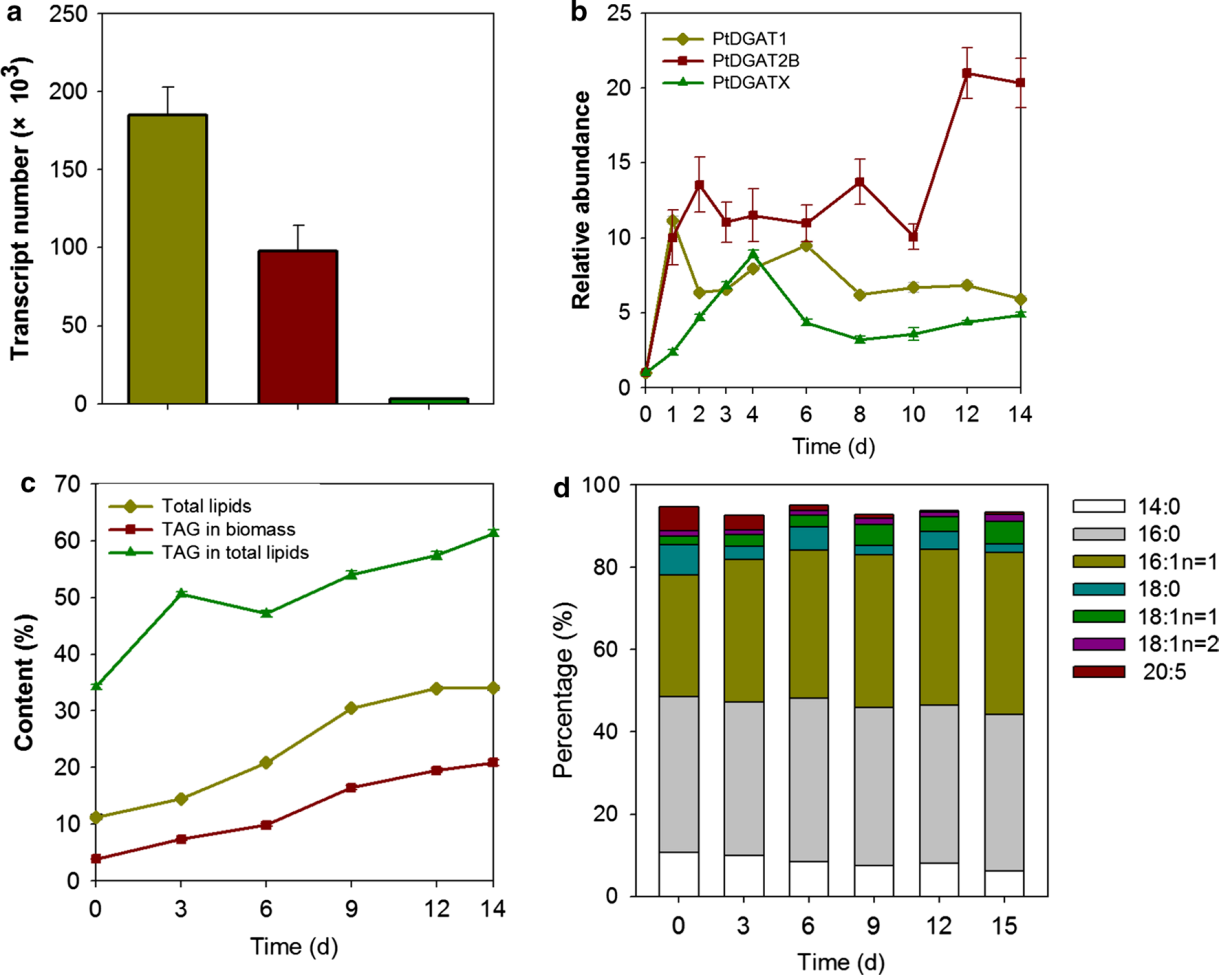
Dynamics of DGATs and lipidomics of *P. tricornutum *following nitrogen depletion. **a** Absolute abundance of DGATs in wild-type *P. tricornutum* at late logarithmic phase. *Y* axis means the transcript number. The concentrations of pMD19T-PtDGAT1, pMD19T-PtDGAT2A, and pMD19T-PtWS/DGAT were determined and diluted to 10^−1^, 10^−2^, 10^−3^, 10^−4^, and 10^−5^ as templates in real-time PCR for standard curve. *R*^2^ with values above 0.99 and amplification efficiencies with values above 90% were obtained for PtDGAT1, DGAT2A, and WS/DGAT genes (amplification curves and melting curves are shown in Additional file 2: Figure S4). **b** Transcriptional dynamics of DGATs upon nitrogen depletion. To impose *N* deprivation, *P. tricornutum* cells in the stationary phase were collected, washed, and re-suspended with *N*-free f/2 medium at room temperature. Samples were collected at indicated periods for transcript analysis. **c** Dynamics of total lipids and TAG in *P. tricornutum* after nitrogen depletion. **d** Profiles of fatty acids in TAG in *P. tricornutum* after nitrogen depletion

**Fig. 3:**
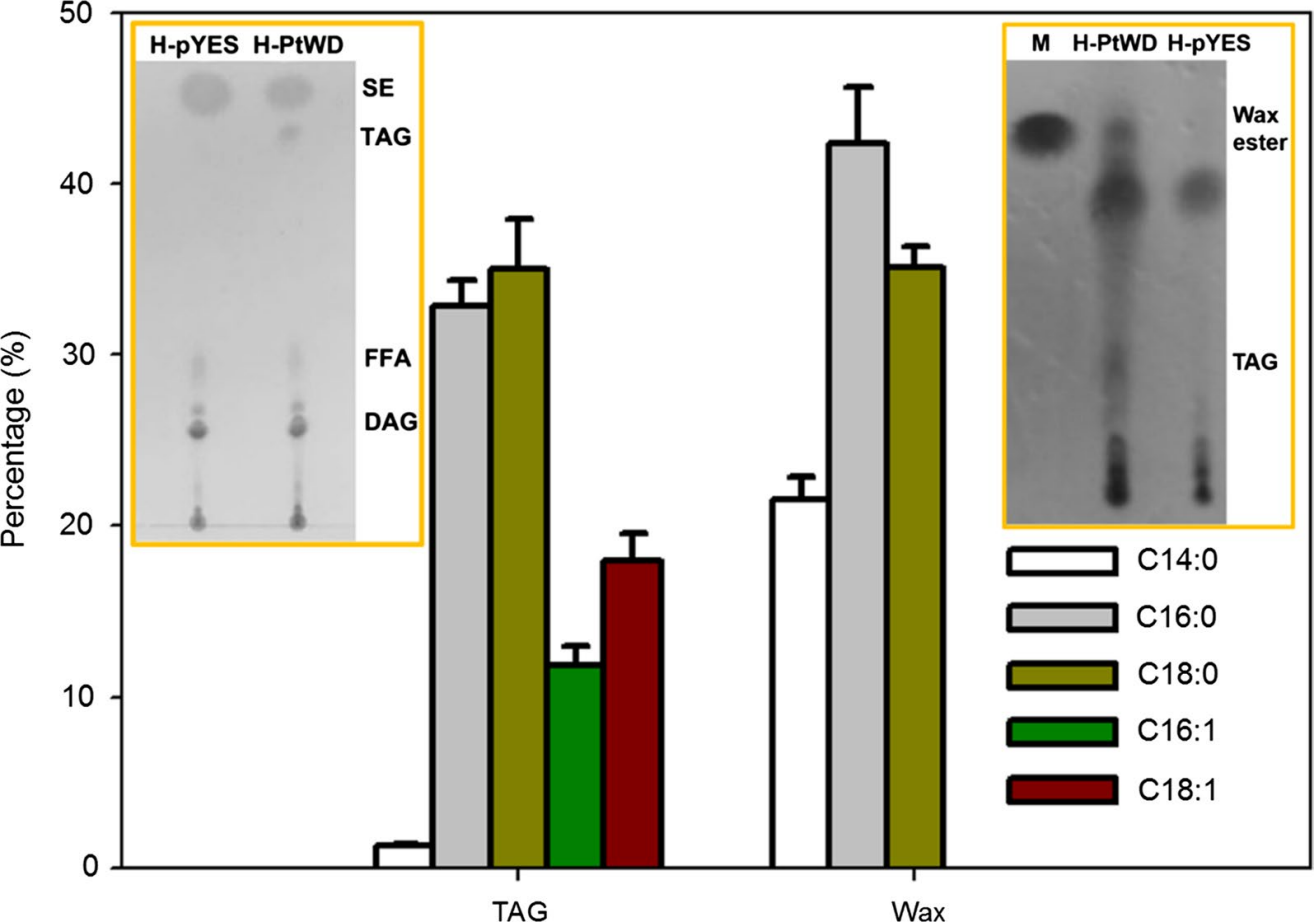
Lipid analysis of the recombinant *S. cerevisiae* H1246. The FA contents of TAG and wax ester were analyzed by GC–MS. *M* cetylpalmitate, *H-PtWD* H1246-PtDGATX, *H-pYES* H1246-pYES2, *SE* sterol ester, *TAG* triacylglycerol, *FFA* free fatty acids, *DAG* diacylglycerol

### PtDGATX, DGAT1, and DGAT2B: Similarities and differences

Two DGAT isoforms, i.e., PtDGAT1 [8] and PtDGAT2B [9], which are essential for TAG formation, have been functionally verified in *P. tricornutum*. The similarities and differences between PtDGATX and other DGAT types were determined via absolute quantitative PCR (qPCR) investigation of the transcriptional dynamics associated with DGAT isoforms in *P. tricornutum*. Among these three DGAT genes, DGAT1 and DGATX exhibited the highest absolute transcript level and the lowest absolute transcript level, respectively, in *P. tricornutum* at late logarithmic phase (Fig. 2a). The three PtDGAT genes were all upregulated under nitrogen (*N*)-depleted conditions, although the expression patterns of the individual DGATs were markedly different. PtDGAT1 showed transient upregulation, with its transcript levels peaking at 1 day following the onset of *N* depletion and declining slightly thereafter (Fig. 2b). PtDGAT2B exhibited the greatest fold change (20.3-fold). Upregulation of PtDGAT2B occurred rapidly within the first day upon *N*− and remained at a relatively constant level thereafter (Fig. 2b). Compared with those of PtDGAT1 and PtDGAT2B (being transcriptionally unregulated rapidly within the first day), the transcript levels of PtDGATX increased more slowly and continuously, peaking at 4 days (Fig. 2b). This suggested that, upon *N*−, relative to PtDGAT1 and DGAT2B, DGATX gene may contribute more to TAG synthesis in the latter stage than during the earlier stages.

The correlation between lipid accumulation and gene regulation (Fig. 2c) was determined via lipidomic analysis. For direct comparison of the results, the same independent batches of cells were used for the transcription and lipidomic analyses. *N*− conditions yielded the following occurrences, namely the (i) total lipid (TL) content increased by approximately twofold (from 11 to 34%) within 14 days (Fig. 2c), (ii) TAG content increased significantly (from 3.85 to 20.87% mg DW^−1^, see Fig. 2c), (iii) FA profiles changed significantly, (iv) content of monounsaturated FAs (i.e., C16:1 *n* = 1 and C18:1 *n* = 1) increased markedly, whereas the content of saturated FAs (i.e., C14:0 and C18:0) and polyunsaturated (i.e., C20:5) FAs decreased (Fig. 2d), and (v) many TAG molecular species increased considerably with the transcript increase of the three DGATs. The lipid composition changed substantially with an increased percentage of TAG in TLs (from 34.22 to 61.33%; Fig. 2c). Regardless of *N* availability, C16:0 and C16:1 in *P. tricornutum* (Fig. 2d) constituted the main FAs of TAG. The transient or progressive upregulation of the DGATs may have resulted in most of the TAG formed during *N* depletion. Therefore, we assumed that the upregulation of these DGAT genes occurs concomitantly with the increase in TAG under *N*− conditions. PtDGAT1 and PtDGAT2B play (in general) a major role in TAG synthesis, whereas PtDGATX may play only a minor role. However, PtDGATX is actively involved in the response to *N*− conditions and appears crucial in the late stage under N− periods, whereas the role of PtDGATX remains unclear.

### Functional rescue of TAG-deficient yeast by PtWS/DGAT

To verify the role of PtDGATX, the gene was expressed in the yeast *Saccharomyces cerevisiae* H1246 that is deficient in TAG biosynthesis. TLC revealed that TAG was absent from the H-pYES strains (H1246 cells harboring an empty pYES2 vector) and a prominent spot corresponding to TAG in the transformants harboring the pYES2-PtDGATX vector (H1246-PtDGATX) was observed (Table 1 and Fig. 3 left inset). Meanwhile, a substantial amount of wax ester was detected in H1246-PtDGATX (Table 1 and Fig. 3 right inset), indicating the dual function of PtDGATX (hereafter PtWS/DGAT) with activities of both WS and DGAT. In H1246-PtWS/DGAT, TAG and wax ester accounted for ~ 10.51 and 5.17%, respectively, of the TLs. C16:1 and C18:1 were the dominant FA species in TLs. However, C16:0 and C18:0 FAs were the principle components of TAG and C14:0, C16:0, and C18:0 FAs were the main FAs in the wax ester (Table 1 and Fig. 3). This indicated that PtWS/DGAT, functioning as either a WS or a DGAT, exhibits a preference on saturated FA substrates in yeast. Moreover, unsaturated FAs (i.e., C16:1 and C18:1 FAs) were detected in TAG, but were absent from the ester, suggesting a difference in substrate preference of PtWS/DGAT functioning as a WS or a DGAT. In addition, the number of TLs accumulated by H1246-PtWS/DGAT was almost triple the number accumulated by the controls (H-pYES strains). This suggested that in vivo manipulating PtWS/DGAT would yield increased lipid production in the oleaginous *P. tricornutum*.

**Table 1:** Fatty acid content of total lipid in *S. cerevisiae*

Fatty acid	H1246-pYES2 (% of TFA)	H1246-PtWS/DGAT (% of TFA)	H1246-PtWS/DGAT (% of TAG)	H1246-PtWS/DGAT (% of wax ester)
C12:0	0.17 ± 0.04	0.36 ± 0.06	–	–
C14:0	0.41 ± 0.11	0.89 ± 0.14	1.33 ± 0.03	21.56 ± 1.29
C16:0	10.81 ± 0.76	16.84 ± 1.15	32.81 ± 1.57	42.37 ± 3.25
C18:0	9.90 ± 0.78	14.36 ± 1.03	35.04 ± 2.86	35.14 ± 1.24
C14:1	0.11 ± 0.01	0.08 ± 0.01	–	–
C16:1	29.93 ± 1.37	25.31 ± 2.14	11.84 ± 1.14	–
C17:1	0.17 ± 0.01	0.04 ± 0.01	–	–
C18:1	47.56 ± 1.95	41.37 ± 2.35	17.97 ± 1.56	–
C18:2	0.11 ± 0.02	0.32 ± 0.01	–	–
C20:1	0.36 ± 0.05	0.34 ± 0.01	–	–
Content	3.25 ± 0.39 (of DW)	9.55 ± 1.21 (of DW; 194%↑)	10.51 ± 0.94 (of TL)	5.17 ± 0.28 (of TL)

*TL* total lipid, *DW* dry weight

### In vivo TAG-synthetic activities of PtWS/DGAT revealed via endogenous overexpression

**Fig. 4:**
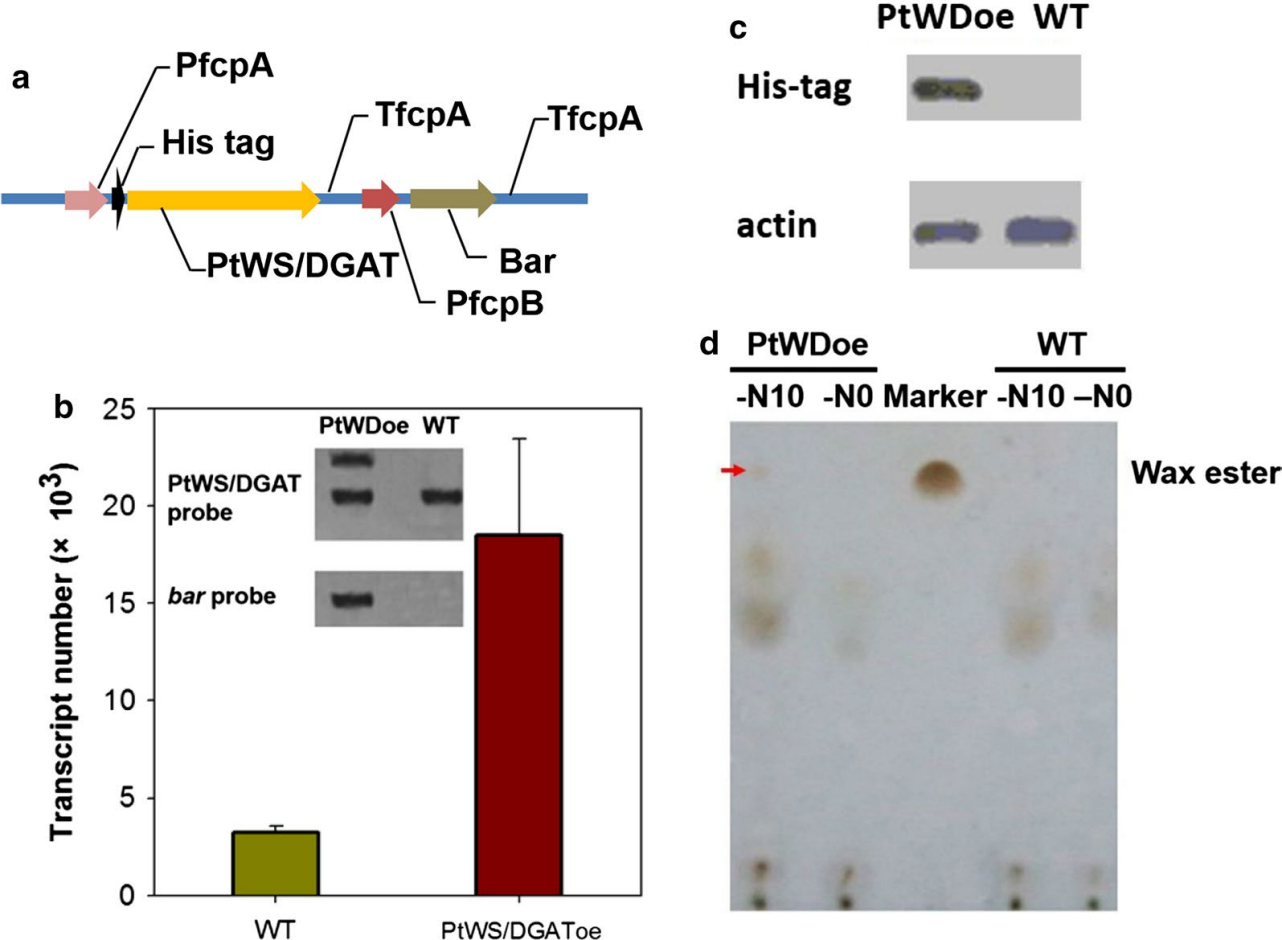
Overexpression PtWS/DGAT in *P. tricornutum*. **a** Construct of the *P. tricornutum* expression vector. PfcpA, promoter of *fcpA* gene; His tag, 6 × His tag; TfcpA, terminator of *fcpA* gene; PfcpB, promoter of *fcpB* gene; bar, the herbicide basta-resistant gene. **b** Quantitative transcriptional levels of PtWS/DGAT in the PtWS/DGAToe and WT cells. Inset, Southern blotting of PtWS/DGAToe with the PtWS/DGAT gene probe or the *bar* gene probe. **c** Immunodetection of PtWS/DGAT-6 × Hisfusion protein with His-tag antibodies. **d** TLC analysis of wax ester in PtWS/DGAToe. *PtWDoe* PtWS/DGAT overexpression *P. tricornutum*, *WT* the wild-type *P. tricornutum*, −*N*0 nitrogen replete culture, −*N*10 nitrogen depletion for 10 days. TAG and wax ester were separated from total lipid by scraping their spots from TLC plates. The FA composition of TAG and wax ester was analyzed by GC–MS

To probe the endogenous function, PtWS/DGAT was overexpressed in *P. tricornutum* (PtWS/DGAToe). The PtWS/DGAT overexpression vector harbored a full-length PtWS/DGAT gene and a basta resistance (*bar*) gene. The PtWS/DGAT gene fused with a His tag was driven by the fcpA promoter (PfcpA) and terminated by the fcpA terminator (TfcpA) region. Moreover, the *bar* gene expression was driven by the fcpB promoter (PfcpB) and terminated with the fcpA2 terminator (TfcpA2) (Fig. 4a). Transformants were selected on *f*/2 plates supplemented with basta. The desired in vivo function of each of these promoters and terminators in *P. tricornutum* was individually validated. Specifically, the transcriptional level of the PtWS/DGAT gene was determined by the absolute quantitative PCR. Under *N*-replete (N+) conditions, a significant increase (4.5-fold) of PtWS/DGAT transcription occurred in the PtWS/DGAToe cells relative to that occurring in the wild-type cells (WT) (Fig. 4b). The integration of the PtWS/DGAT vector was validated via Southern blotting, as indicated by a clear band using *bar* gene as a probe (Fig. 4b inset). Using the PtWS/DGAT gene as a probe during blotting revealed two bands in PtWS/DGAToe and a single band in WT (Fig. 4b inset). This was consistent with a single integration of the exogenous PtWS/DGAT gene into the alga chromosome. Furthermore, in vivo expression of the PtWS/DGAT protein was validated by using His-tag antibodies to detect the PtWS/DGAT-6 × His fusion protein via immunoblotting. A band was detected in PtWS/DGAToe, whereas no signal was detected in WT (Fig. 4c).

Compared with those of WT, the respective TL content and TAG content of the PtWS/DGAToe increased by (i) 21 and 40% under *N*+ conditions and (ii) 10 and 25% following *N*− (see Table 2). The levels of unsaturated FAs (e.g., C16:1 *n* − 1, C16:1 *n* − 2, and C18:1 *n* − 1 FAs) decreased and the levels of saturated FAs (especially the C14:0, C16:0, and C18:0 FAs) increased slightly under both *N*+ and *N*− conditions (Table 2). This may have resulted from the substrate preference of PtWS/DGAT on saturated FAs. Therefore, regardless of *N* availability, PtWS/DGAT exhibited a DGAT activity with a preference on saturated FAs. In contrast, under *N*−, the WS activity of PtWS/DGAT seemed to be higher in the late stage (than in the early stages). Wax ester was absent from either WT or the PtWS/DGAToe cells under *N*+ conditions. In contrast, following prolonged *N*−, a small amount (0.53 ± 0.05% of TLs) of wax ester occurred in the PtWS/DGAToe, but was absent from WT, although a large amount of lipids was loaded for TLC analysis (Fig. 4d). Hence, although the activity as WS is condition dependent and relatively minor, PtWS/DGAT exhibits activities in both wax ester synthesis and TAG synthesis in *P. tricornutum*.

**Table 2:** Fatty acid content of TAG in the recombinant *P. tricornutum*

Fatty acid	Nitrogen repletion		Nitrogen depletion for 10 days	
	Wild type (% of TAG)	PtWS/DGAToe (% of TAG)	Wild type (% of TAG)	PtWS/DGAToe (% of TAG)
Saturated fatty acid				
C14:0	10.73 ± 0.83	11.07 ± 0.64	6.27 ± 0.23	7.36 ± 0.58
C15:0	0.59 ± 0.05	0.72 ± 0.07	0.52 ± 0.03	0.46 ± 0.04
C16:0	32.84 ± 2.45	34.47 ± 3.49	40.04 ± 3.39	43.82 ± 2.93
C18:0	7.31 ± 0.34	8.01 ± 0.48	3.07 ± 0.04	5.31 ± 0.21
C19:0	1.26 ± 0.21	1.71 ± 0.30	0.72 ± 0.01	0.84 ± 0.03
C20:0	0.31 ± 0.02	0.39 ± 0.03	0.19 ± 0.01	0.23 ± 0.01
C22:0	0.83 ± 0.07	0.81 ± 0.03	0.57 ± 0.03	0.52 ± 0.05
C24:0	3.73 ± 0.39	4.01 ± 0.23	3.24 ± 0.27	2.12 ± 0.72
Monounsaturated fatty acid				
C16:1 *n* − 1	29.56 ± 2.32	28.06 ± 3.74	36.85 ± 2.46	34.25 ± 2.93
C16:1 *n* − 2	1.11 ± 0.32	0.73 ± 0.06	1.53 ± 0.17	1.20 ± 0.08
C18:1 *n* − 1	2.11 ± 0.34	1.71 ± 0.25	4.43 ± 0.48	2.34 ± 0.40
C18:1 *n* − 2	1.25 ± 0.04	1.27 ± 0.34	0.82 ± 0.02	0.17 ± 0.01
C24:1	0.27 ± 0.01	0.28 ± 0.02	0.39 ± 0.03	0.13 ± 0.01
Polyunsaturated fatty acid				
C16:3	1.55 ± 0.06	1.04 ± 0.05	0.48 ± 0.03	0.53 ± 0.09
C20:3	0.33 ± 0.02	0.13 ± 0.01	0.20 ± 0.01	0.15 ± 0.01
C20:4	0.27 ± 0.01	0.21 ± 0.01	0.18 ± 0.01	0.20 ± 0.01
C20:5 (EPA)	5.86 ± 0.11	4.59 ± 0.07	0.46 ± 0.01	0.37 ± 0.01
TAG (% of DW)	3.85 ± 0.22	5.39 ± 0.15 (40%↑)	18.27 ± 1.48	22.78 ± 1.59 (25%↑)
Total lipid (% of DW)	11.25 ± 1.17	13.61 ± 1.84 (21%↑)	32.64 ± 2.51	36.18 ± 2.38 (10%↑)

*DW* dry weight, *EPA* eicosapentaenoic acid, *TAG* triacylglycerol, ↑ increased

### Engineering PtWS/DGAT for increased lipid productivity

**Fig. 5:**
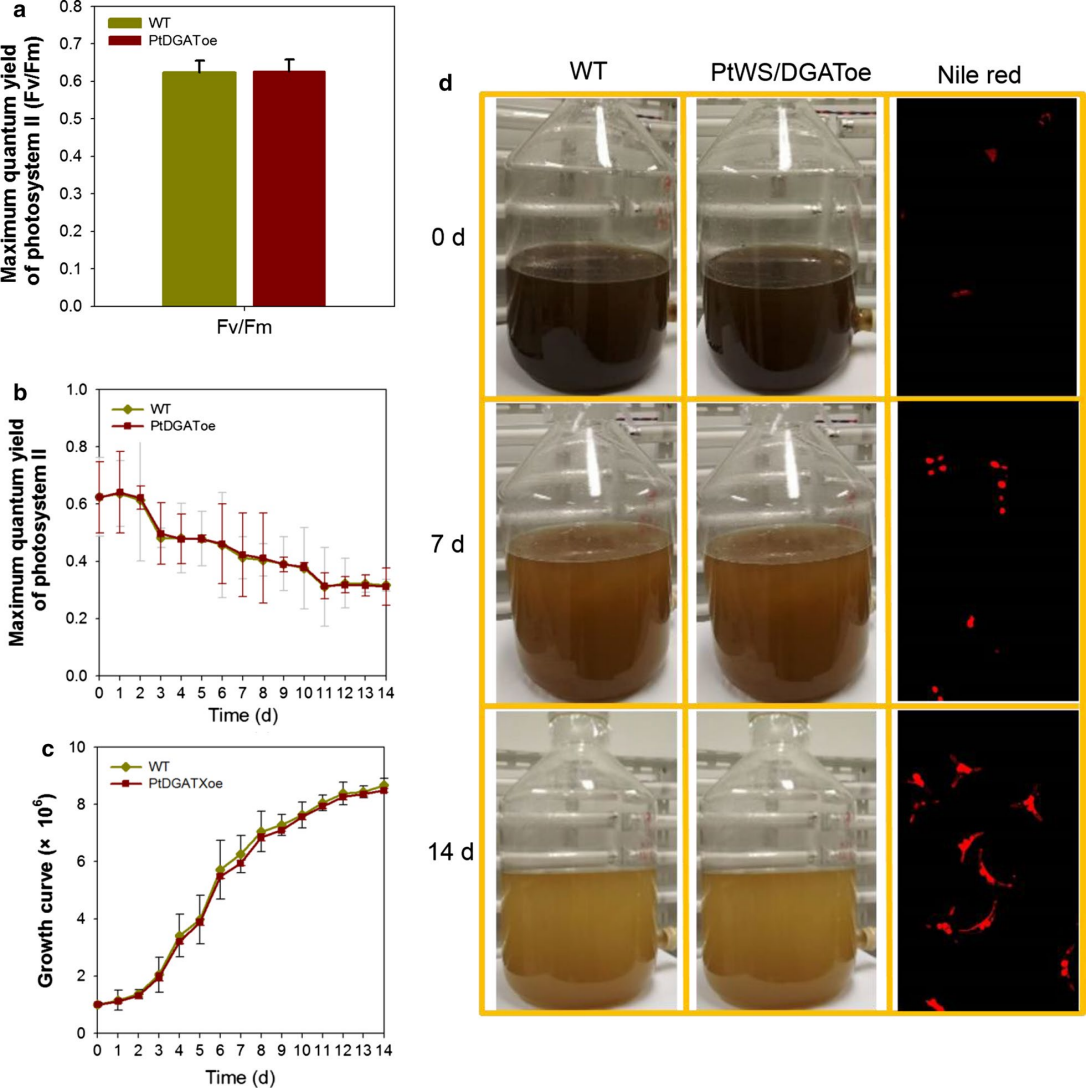
Assessment of the production performance of PtWS/DGAToe. **a** Typical results of photosynthetic measurements of WT and the PtWS/DGAToe strains under nitrogen repletion conditions. **b** Comparison of maximum quantum yield of photosystem II of WT and the PtWS/DGAToe strains under nitrogen depletion conditions. **c** Growth of WT and the PtWS/DGAToe lines under nitrogen repletion conditions. **d** Image of WT and the PtWS/DGAToe lines following nitrogen depletion in a 10-L photobioreactor. Only typical results of Nile red staining are shown in the right column since no significant difference is observed between WT and the PtWS/DGAToe cells. *PtWS/DGAToe*, PtWS/DGAT overexpression *P. tricornutum*

The in vitro and in vivo evidence indicated that perturbation on PtWS/DGAT resulted in increased production of TLs and TAG. However, increased cellular lipid biosynthesis occasionally leads to decreased growth, thereby neutralizing the overall lipid productivity [2]. Biomass accumulation (i.e., growth rate of microalgal strains) is thus crucial for determining the lipid productivity. Therefore, to determine whether the genetically modified (GM) *P. tricornutum* cells can be exploited for enhanced lipid production, the photosynthetic performance of both WT and PtWS/DGAToe was tracked under either *N*+ or 14-day *N*− conditions. Under *N*+ conditions, both lines demonstrated a high *Fv/Fm* value (the maximum quantum yield of photosystem II, approximately 0.62, which implies a fit growth condition; a typical result is shown in Fig. 5a). Upon nitrogen depletion, although *Fv/Fm* values of both lines were decreased, barely any difference was observed between WT and the GM strains (Fig. 5b and Additional file 3 Table S2). Consistently, the growth of the two lines was very similar under the explored conditions (monitored by detecting cell numbers daily; see Fig. 5c for growth under *N*+ conditions and see Additional file 2: Figure S5 for that under both *N*+ and *N*− conditions). Moreover, the dry weight (DW) of PtWS/DGAToe strains (397.50 ± 16.25 mg L^−1^) was determined after fourteen-day nitrogen starvation which was similar to that of WT (398.75 ± 26.25 mg L^−1^) and the DW of both was comparable to the levels of documented *P. tricornutum* strains (Table 3) [38].

**Table 3:** Productivity comparison of *P. tricornutum* WT and PtWS/DGAToe lines cultured in a 10-L PBR following nitrogen deprivation for fourteen days

	Wild type (mg L^−1^day^−1^)	PtWS/DGAToe (mg L^−1^day^−1^)
Dry weight	28.5 ± 1.9	28.4 ± 1.2 (0.3%↓)
Total lipid	9.3 ± 0.9	10.5 ± 0.6 (13.5%↑)
Triacylglycerol	5.4 ± 0.1	6.4 ± 0.3 (18.4%↑)
Wax ester	0	0.8 ± 0.1 (↑)

↑ increased, ↓ decreased

Combined with the elevated lipid and TAG content, this indicated that GM *P. tricornutum* cells were quite capable of photoautotrophic growth, absorbing CO_2_ and producing (especially) biomass, lipids, and TAG. The stability and scalability of the GM *P. tricornutum* were further determined by evaluating the life cycle performance in a 10-L photobioreactor (PBR) (Fig. 5d). The growth rate of the mutant under nitrogen repletion conditions was identical to that of WT, reaching a biomass productivity of 28.4 mg L^−1^ day^−1^ (See “Methods” for details). The biomass and lipid content were determined for both WT and GM *P. tricornutum* cultivated in nitrogen-free *f*/2 medium in PBR for 14 days. Compared with those of WT, the respective TL content and TAG content of the PtWS/DGAToe increased by 13.8 and 18.4% (371.1 mg g^−1^ DW TL and 226.4 mg g^−1^ DW TAG in PtWS/DGAToe) (Table 3), corresponded to a TAG productivity of 6.4 mg L^−1^ day^−1^ (an 18.4% increase relative to that of 5.4 mg L^−1^ day^−1^of WT). Moreover, following prolonged *N*− conditions, a considerable amount of wax ester (from undetectable levels to 28.3 mg g^−1^ DW) was also generated. Although the productivity remains to be improved, our results provide insight into the customized production of different high-value chemicals via engineering of a single gene [39].

## Conclusions

We have discovered in diatom *P. tricornutum* a dual-function WS/DGAT which has previously only been characterized in bacteria and plants. Distinctive to DGAT1s, DGAT2s, and DGAT3s, PtWS/DGAT exhibited activities of both wax ester and TAG biosynthesis. Moreover, PtWS/DGAT, functioning as either a WS or a DGAT, exhibited a preference on saturated FA substrates. Overexpression of PtWS/DGAT endogenously yielded wax under *N*− conditions and increased production of TLs and TAG (relative to WT) under both *N*+ and *N*− conditions. Scale-up cultivation of GM diatom strains yielded increased production of TLs, TAG, and wax esters, without compromising cell growth. These findings improve our understanding of the origin, function, and cellular properties of DGATs in TAG biosynthesis and have important implications for the rational engineering of microalga-based oils and waxes for future industrial use.

## Methods

### Strains and growth conditions

The *P. tricornutum* strains were kindly donated by Prof. Mingyan Yin of the Key Laboratory of Experimental Marine Biology, Institute of Oceanology, Chinese Academy of Sciences. *P. tricornutum* was grown as previously described [3], i.e., at 23 °C under a 40–50 μmol photons m^−2^ s^−1^ light with a 12/12 h light:dark photoperiod. To impose N deprivation, *P. tricornutum* cells in the stationary phase (8.5 × 10^6^ cell mL^−1^) were collected, washed, and re-suspended in nitrogen-free *f*/2 medium. Samples were collected at indicated periods for transcript and lipid analysis [40]. For the scaled up cultivation of the diatom, 10-L cylinder glass bottles (50 cm diameter and 70 cm height) were used. Cells with an initial concentration of 5 × 10^6^ cell mL^−1^ were cultured using a continuous filtered gas at 1.3 m^3^ min^−1^ and 40–50 μmol photons m^−2^ s^−1^ illumination with a 12/12 h light:dark photoperiod. The biomass productivity was calculated 7 days later using the following formula:

$$ {\text{Biomass productivity}} = \frac{\text{DWs}-{\text{DWi}}}  {T \times V}, $$

where DWs is the DW of cells at sampled time point, DWi is the DW of initial culture, *T* is the duration of culture period, and *V* is the volume of algal culture. Afterward, cells were collected, washed, and re-suspended in nitrogen-free *f*/2 medium. Lipid measurements were conducted after 14 days.

#### Phenotyping and Nile red staining

The growth of microalgae was monitored by measuring the turbidity (OD_750_) or cell number at indicated intervals. The chlorophyll fluorescence parameter was measured with Diving PAM (WALZ, Germany). The lipid accumulation was monitored by Nile red staining. *P. tricornutum* cultures (approximately 4 × 10^6^ cells) at indicated time points were harvested, stained with 1% (v/v) Nile Red (0.5 mg mL^−1^ in dimethylsulfoxide), incubated at room temperature for 5 min, and immediately observed by Laser scanning confocal microscopy with an excitation filter at 480 nm (FluoViewFV1000, Olympus, Japan).

### Sequence analysis of *PtWS/DGAT*

Sequence analysis and comparison were undertaken in accordance with previous reports [41]. In brief, sequence alignment and phylogenetic analysis were performed with BLAST (http://www.ncbi.nlm.nih.gov/BLAST) and CLUSTALW, respectively [42]. Bootstrap support values were estimated using 100 pseudo-replicates. The Conserved Domain Database (CDD; http://www.ncbi.nlm.nih.gov/cdd/), Pfam (http://pfam.sanger.ac.uk/search) and SMART (http://smart.emblheidelberg.de/) databases were used to search for structurally conserved domains within specific proteins. The transmembrane region was analyzed by the software TMHMM (http://www.cbs.dtu.dk/services/TMHMM/), DAS (http://www.sbc.su.se/~miklos/DAS/), and TMpred (http://www.ch.embnet.org/software/TMPRED_form.html). The complete amino acid sequence of PtDGATX protein was submitted in the software online and the probable transmembrane regions were shown.

### Gene expression analysis

Genomic DNA, total RNA extraction, and reverse transcription were performed as previously described [3, 40, 41]. Total RNA was extracted and purified with the Eastep Super Total RNA Extraction Kit (Promega) and then subjected to genomic DNA digestion and reverse transcription using a PrimeScript RT reagent Kit with gDNA Eraser (Takara) [40, 41].

Relative qPCR was performed in two steps as given in our earlier description [40]. First, 0.5 μg RNA was mixed with 2 μL of 4×*g* DNA wiper Mix and Nuclease-free H_2_O was added to a final volume of 8 μL. The mixture was kept at 42 °C for 2 min. Second, 2 μL of 5 × HiScript II Q RT SuperMix IIa was added to the above mix. Reverse transcription (RT) reactions were performed in a GeneAmp^®^ PCR System 9700 (Applied Biosystems, USA) with a program of 25 °C for 10 min, 50 °C for 30 min, and 85 °C for 5 min. The 10 μL RT reaction mix was then diluted with 10 μL nuclease-free water and held at − 20 °C. Real-time PCR was performed using LightCycler^®^ 480 II Real-time PCR Instrument (Roche, Swiss) with 10 μL PCR reaction mixtures including 1 μL of cDNA, 5 μL of 2 × QuantiFast^®^ SYBR^®^ Green PCR Master Mix (Qiagen, Germany), 0.2 μL of forward primer, 0.2 μL of reverse primer, and 3.6 μL of nuclease-free water. Reactions were incubated at 95 °C for 5 min, followed by 40 cycles of 95 °C for 10 s and 60 °C for 30 s. At the end of the PCR cycles, melting curve analysis was performed. Each sample was run in triplicate. The expression levels of mRNAs were calculated using the 2^−ΔΔCt^ method. The mRNA expression level was normalized using the 18S gene as the internal control [43].

For absolute qPCR, RT reaction was conducted following the instruction of Fast Quant RT Kit KR106 (with gDNase; Tiangen, China). The total RNA (500–1000 ng) in 8 μL RNAase-free H_2_O was preheated in 65 °C for 5 min and transferred to ice immediately, mixed with 2 μL 5×*g*-DNA buffer (containing g-DNase) in 42 °C for 3 min, and transferred to ice immediately. The solution was then mixed with 10 μL premixed buffer containing primers and FastQuant RT Enzyme. RT reaction was performed in a GeneAmp^®^ PCR System 9700 (Applied Biosystems, USA) at 42 °C for 15 min, 95 °C for 3 min, and 4 °C for 10 min. PtDGAT1, PtDGAT2A, and PtWS/DGAT genes were amplified and cloned into pMD19T vector (TAKARA, China). The concentrations of vectors pMD19T-PtDGAT1, pMD19T-PtDGAT2A, and pMD19T-PtWS/DGAT were measured with Nanodrop ND2000 (Thermo, USA), and diluted to 10^−1^, 10^−2^, 10^−3^, 10^−4^, and 10^−5^ as templates for standard curve. The qPCR was performed using ABI7500 (ABI, USA) with a 25 μL PCR reaction mixture (included 1 μL of cDNA, 12.5 μL Bestar SybrGreen Qpcrmaster mix (DBI Bioscience, Germany), 0.25 μL of forward primer, 0.25 μL of reverse primer, and 11 μL of nuclease-free water). Reactions were incubated at 95 °C for 5 min, followed by 40 cycles of 95 °C for 10 s and 60 °C for 30 s. All primers were validated by PCR, sequencing, and gel electrophoresis where a single and clear band was observed for each pair of primers (Additional file 2: Figure S4a). Amplification curves and melting curves are shown in Additional file 2: Figure S4b. As for absolute qPCR, *R*^2^ with values above 0.99 and amplification efficiencies with values above 90% were obtained for PtDGAT1, DGAT2A, and WS/DGAT genes (Additional file 2: Figure S4c).

### Gene cloning and expression in *S. cerevisiae*

*Saccharomyces cerevisiae* strain H1246 (relevant genotype: MATa ADE2-1can1–100 ura3-1 are1-D::HIS3 are2-D::LEU 2 dga1-D::KanMX4 Iro1-D::TRP1 containing knockouts of DGA1, LRO1, ARE1, and ARE2) was kindly donated by Prof. Sten Stymne (Scandinavian Biotechnology Research, Alnarp, Sweden). *S. cerevisiae* H1246 was maintained in a YPD medium at 30 °C and manipulated as previously described [44, 45]. PtWS/DGAT cDNA was cloned and inserted into the yeast expression vector pYES2 (Invitrogen). The recombinant PtWS/DGAT constructs were transformed into the TAG-deficient *S. cerevisiae* strain H1246 (Δdga1Δlro1Δare1Δare2) using an *S. cerevisiae* EasyComp TM transformation kit (Invitrogen), as described in our previous studies [44, 45]. Prior to transformation, yeast cells were cultivated in 1% (w⁄v) yeast extract, 2% (w⁄v) peptone, and 2% (w⁄v) glucose (YPD medium) at 30 °C. H1246-pYES cells harboring the empty pYES2 vector were used as negative controls. Transformants were selected via uracil prototrophy on a yeast synthetic minimal-defined medium (SC) lacking uracil (Invitrogen). For functional expression, a minimal selection medium containing 1% (w⁄v) raffinose and 2% galactose (w/v) was inoculated with the PtWS/DGAT transformants and incubated at 22 °C for 3–5 days. The induced cells were tested for the presence of PtWS/DGAT by PCR, and its expression by RT-PCR (see Additional file 3: Table S1 for PCR primers). Cells with positive results (H1246-PtWS/DGAT) were harvested via centrifugation, washed twice with 0.1% (w⁄v) NaHCO_3_, freeze-dried, and used for lipid analysis.

### Expression vector design and *P. tricornutum* transformation

The PtWS/DGAT overexpression vector harbored a full-length PtWS/DGAT gene and a basta resistance (Bar) gene, each of which was driven by an endogenous promoter and terminated by an endogenous terminator (Fig. 4a). The PtWS/DGAT gene was driven by the Fucoxanthin-chlorophyll protein A promoter (PfcpA) and terminated by the Fucoxanthin-chlorophyll protein A1 termination (TfcpA) region. Expression of the Bar gene was driven by the Fucoxanthin-chlorophyll protein B promoter (PfcpB) and terminated with TfcpA terminator. The fragments were cloned with primers harboring indicated restriction enzyme cutting sites (Additional file 3: Table S1), while the commercial vector pBluescript SK(+) was employed as an acceptor vector. The PtWD/DGAT gene was inserted into the *Xba*I and *Sac*II sites, while the PfcpA fragment and the TfcpA fragment were inserted into the position between the *Sal*I and *Hind*III sites and between the *Sma*I and *Bam*HI sites, respectively. On the other hand, the bar gene was inserted into the *Eco*RV and *Pst*I sites, while the PfcpB fragment and the TfcpA2 fragment were ligated into the *Spe*I and *Xba*I sites and *Sac*II and *Sax*I sites, respectively.

Bombardment transformation was performed using a GJ-1000 apparatus (Ningbo, China) with a field strength and shunt resistance of 1350 psi and 9 cm, respectively. Afterward, the cells were immediately transferred onto *f*/2 plates with 15 μg mL^−1^ basta and 150 μg mL^−1^ kanamycin, and incubated at 23°C with continuous illumination of 40–50 μmol photons m^−2^ s^−1^ until transformants were observed. Individual colonies were picked and transferred into fresh *f*/2 plates with selective agents. The transformants were subjected to successive passages and maintained in a liquid *f*/2 medium.

### Immunodetection

The stable integration of the transforming cassette into the chromosome and expression were validated via Southern blotting and western blotting, respectively. For Southern blotting, no less than 1 μg genomic DNA was digested with *Hind*III and analyzed by DNA gel blotting using nonradioactive DIG-containing PtWS/DGAT or Bar gene probes (PCR DIG probe synthesis kit; Roche Diagnostics). The PtWS/DGAT or Bar gene probes were labeled with DIG-dUTP using corresponding primers (Additional file 3: Table S1). His-tag antibodies were used to detect the expression fusion protein of PtWS/DGAT-His-tag. To determine whether PtWS/DGAT has a transmembrane motif, the PtWS/DGAToe cells at the log phase were harvested and soluble and membrane proteins were used for (previously described) immunodetection [37]. In brief, cell pellets were re-suspended in 10 mL extraction buffer (50 mM Tris-HCl, pH 8.0, 1 mM EDTA, 10 mM KCl, 1 mM MgCl_2_, 1 mM β-mercaptoethanol, 0.1 mM phenylmethylsulfonyl fluoride, 1 mg mL^−1^ leupeptin, and 0.25 M sucrose) and broken by an ultrasonic cell disrupter (SCIENTZ-ID, China) at 100 W for 20 min. The soluble and membrane proteins were separated via centrifugation at 18,000 *g* for 20 min (Beckman Optimatm L-100 XP, America). Afterward, each protein was immunoblotted with His-tag antibodies. For each sample, 35–40 μg protein was loaded and the Coomassie staining is shown in Additional file 2: Figure S6.

### Lipid extraction and analysis

Cells were harvested via centrifugation at 4 °C and 5000* g* min^−1^ for 5 min, freeze-dried, and used for lipid analysis. Lipid extraction and TLC analysis of the neutral lipids were performed as described by Yoon [7]. For TLC analysis, a mixture of normal hexane/diethyl ether/acetic acid (70/30/1 by volume) was used as the mobile phase for TAG analysis. Wax ester analysis was performed using petroleum ether/diethyl ether/acetic acid (90:10:1). TAG and wax ester were detected by iodine vapor and 50% sulfuric acid, respectively. The lipid quantification was performed by GC–MS, as previously described [14, 46].

#### Statistical analysis

To ensure reproducibility, the experiments were all performed with at least three biological triplicates. Each of the presented values corresponds to a mean ± SD. Statistical analyses were performed using the SPSS statistical package (http://www-01.ibm.com/software/analytics/spss/). Furthermore, the paired-samples *t* test was performed and differences were considered statistically significant at *P* values of < 0.05.

## Additional files

**Additional file 1.** PtWS DGAT cDNA sequence.

**Additional file 2:** **Figure S1.** Phylogenetic analysis of DGATXs. **Figure S2.** Multiple protein-sequence alignments of DGATXs. **Figure S3.** Representative topology of DGAT1s, DGAT2s, DGAT3s and WS/DGAT. **Figure S4.** Assessment of primers of PtDGAT1, DGAT2A, and PtWS/DGAT genes for qPCR. (a) Gel electrophoresis of the PCR product using PtDGAT1, DGAT2A and PtWS/DGAT primers. (b) Amplification curves and melting curves of PtDGAT1, DGAT2A and PtWS/DGAT primers. (c) Standard curve for absolute qPCR using vectors pMD19T-PtDGAT1, pMD19T-PtDGAT2A and pMD19T-PtWS/DGAT as templates. The vector concentrations were determined by Nanodrop ND2000 and diluted to 10^−1^, 10^−2^, 10^−3^, 10^−4^, and 10^−5^ for standard curve. R^2^ with values above 0.99 and amplification efficiencies with values above 90% were obtained for PtDGAT1, DGAT2A and WS/DGAT genes. **Figure S5.** Growth of WT and the PtWS/DGAToe lines under nitrogen repletion conditions (black bar) followed by nitrogen starvation (white bar).

**Additional file 3:** **Table S1.** Primers used in this study. **Table S2.** The *Fv*/*Fm* of WT and PtWS/DGAToe *P. tricornutum* lines following nitrogen deprivation for fourteen days.

## Abbreviations

*bar*: a basta resistance gene
DAG: diacylglycerol
DGATs: acyl-CoA:diacylglycerol acyltransferases
DW: dry weight
FAs: fatty acids
GC–MS: gas chromatography–mass spectrometry
GM: genetically modified
H-pYES strains: H1246 cells harboring an empty pYES2 vector
H1246-PtDGATX: the H1246 transformants harboring the pYES2-PtDGATX vector
*N*−: *N*-deplete conditions
*N*+: *N*-replete conditions
PBR: photobioreactor
PCR: polymerase chain reaction
PfcpA: a fcpA promoter
PfcpB: a fcpB promoter
TAG: triacylglycerol
TfcpA: a fcpA terminator
TfcpA2: a fcpA2 terminator
TL: total lipid
WS: wax ester synthase
WT: wild type
