# Supplementary material for: Characterization and engineering of a dual-function diacylglycerol acyltransferase in the oleaginous marine diatom Phaeodactylum tricornutum
Source: Biotechnol Biofuels. 2018 Feb 9;11:32. doi: 10.1186/s13068-018-1029-8 (PMC5806285; doi:10.1186/s13068-018-1029-8)
Supplement: Supplementary file 1 — Additional file 1. PtWS DGAT cDNA sequence. [file 13068_2018_1029_MOESM1_ESM.docx]

**PtWS/DGAT cDNA sequence**

The nucleotides in blue represent the 5’-UTR region of *PtWS/DGAT* and the ones pink-highlighted are the 3’-UTR region. The start codon and stop codon are yellow highlighted.

AGTTTGTTCCTGTTTTCGTGTCTTGACATGGAATCACAGAGAACCTGAGCCCCACACGATAGATATTCGATGGGAATTCACTATTGGAAGCCGGCCTCGATTGACGCAAAATCACTTATCCGGCCAAAATGTAGAGATTGGCTGTGACTGTGAAAGTGAGTAACAAAGGTAGGGAGATCCTCTTTTTGCAGCGAAAACCCCCTGTATTTCGAATATTTAAACACATTCAAAACAGATTTTGTCTGGATGACCTACCCCTACGATACGTAAAAAGGAAACGTCTTTCTGATTGGGTAGATCCTGCTTCAGACTTGACGTATTGATGTCGCTCGGATTCCTATCAGTTTGACCTCGACAACCGAAAGCAAATTTGGATTTGCTACGTCTTGGTGTCACAGTCAATCTTTGCCCATTCGAGAAAAAGTTCTCTATTTTTTGGTGTGCAATCAACCCGAATATCCGAGGAACGAACCAAGAGTCATGGATGTCTTTGGCAGCAAATTGGTTGAAGCGCTCTCCGTCTATCATGCTTCCTGGCTGCTTGGTTTAAGCGTCACAGTAGCGGTAGCGATCGCAATCAAAATGTCCTCCAATCAAAAAAGTCGCTCGCCTCTGCACCGAAAGTTTTCTTTCACATCCGTCGGAATGGCCATCGGAATCTTCCCCGAATCGGTCAAAGCTCCCACAACAATCATCAACGCGGCAATCTACTTTTCAACATGTCCCGCGGAGAAGGATCTCATTGAACTGGCGGTAAAACCTATGCTTGCTTTCACGCGACTGTCAACGATTCCTGTCCCGGAAACGGCCAACTGCCGACCTTCCACGCGGTCTTTTGCGCCATCGGAACTCATTCGGAAGGTTGAAATATCAGGTAAATGCATCAAGTCGACAAATGATGTCATATTTAAGCACCTGCAAGAGTCGCTCTCGACAGAGCGAGACGATTTGCCGTGGTGGGAGTTTCTAGTGGTCGAAAACGTTGGCGAGGGCGAGTCTGCCGTCGTTCTACGGATGCACCACGCCCTAGCGGATGGTATTTCGCTAGTACACGTTTTTGAAAAGTTCATAACCTACGAAGATGGTTCGCCGGTTTTGTCCATTATTCTGTCCAACATGGCGCAGAAGAGCAAAGTCGAGAAAACGCACAAAACAAATCCCTTCCGCCTTGCTTGGATGCTTGTCCGAGATGCTACCAAGGTCCTCACGTTGGGTCTTTCGCGTTCGGACGATCCCACTATCTTTACCGAACCGAATCAGACGTATGTGCATTCGCAGCATCGAGAATGTGTGGTTTTCCCAACGTTTTCATTGGCCTTCGTTAAGCGGCTGAAAACAGCAGCCAACGTGACCGTTAACGATATTCTCATGACCGCGGTCAGCCAAGCGGTACACGAGTACTGCCGAGCTGAATCCTGCTCGGTCTTGATGGGAAAAGGAGCATCGCTTCAGTCACGTGCATTATTGCCGATAGCGTTGCCGCGATCCGCGTCAGACTTGGAACATCCTTCCACGGCTTTGCGCAACAAGTGGTGTCTTGTTTCGGCAAATATGAGCATTGGCTGTGTCGACCTAGTGGATCGTCTTAATTCGATCCACCAGACTACTGTTCACTTAAAAGGAAGCCCAATTGCCATGGTCCAACTCAGTCTGCAAAACAAATTGGCAAGTCGATTGCCTAAAATAGTCGCTCGACAAACCATGCTGGACATTTTTCGAAGGCATTCGCTTGTCTTTTCCAACGTTCCCGGCCCAGATCGTCCGTGTCAATTGGCCGGGCAAACAGCCACTGGAGTACAAATGTTCTATAGCAACCTGATTCCTCAAGTTGGATTGCTGTCGTACGCCGGGAACATTTACGGTAATATAGTCCTAGACACTGGTGCCGTGCCCAACGCTGAATCTTTGGCTGGCCATTACGCAAAGGCGCTTGTCGACATGGCGACCCTGCTCAACGTCGACAAAATTCCAACGAATTTACAGTCGTACTTCTAAGGATGATACCCACGGTCCCTGTGCAAGTTTTTAAGATAGCCTCATTCATACAAACCTCTTCAAGTTAACGCATGGTTAAAGGAAGAAGCCTATCTGCCCATGCTATACTACTAGAGTGTCGAAAGCATCCCCAATTTCTTGTGACTTCCCGTTGGATTCGTCAACCAAAGTTCATGTCGTAAGGTGCAGGGTAAAACACTGCATGTTGATTTTTATAGAGAATCCATTCCGAAGAAGGTTTCCACCTTAAATTGAACTACCTCGTTCTCTGAGCAACAAATTGGATTAGAGGTCGACCATGATCGCTATCAACGTGCACCCTATCATGAAGAATCGACCAAGCTTTGTACCCCTGCTGCTGGAAAATGTTTGACAATTCAGGACGCATCAAAACATGCTTTGGCTGACGAGATCCAAAAAAAATAACTCTTTAGTGTCAATACGCAATTTCTGGCAATCTCCACCGACTCCAGCA
