# Supplementary figures and images for: Characterization and engineering of a dual-function diacylglycerol acyltransferase in the oleaginous marine diatom Phaeodactylum tricornutum
Source: Biotechnol Biofuels. 2018 Feb 9;11:32. doi: 10.1186/s13068-018-1029-8 (PMC5806285; doi:10.1186/s13068-018-1029-8)

## Slide 1
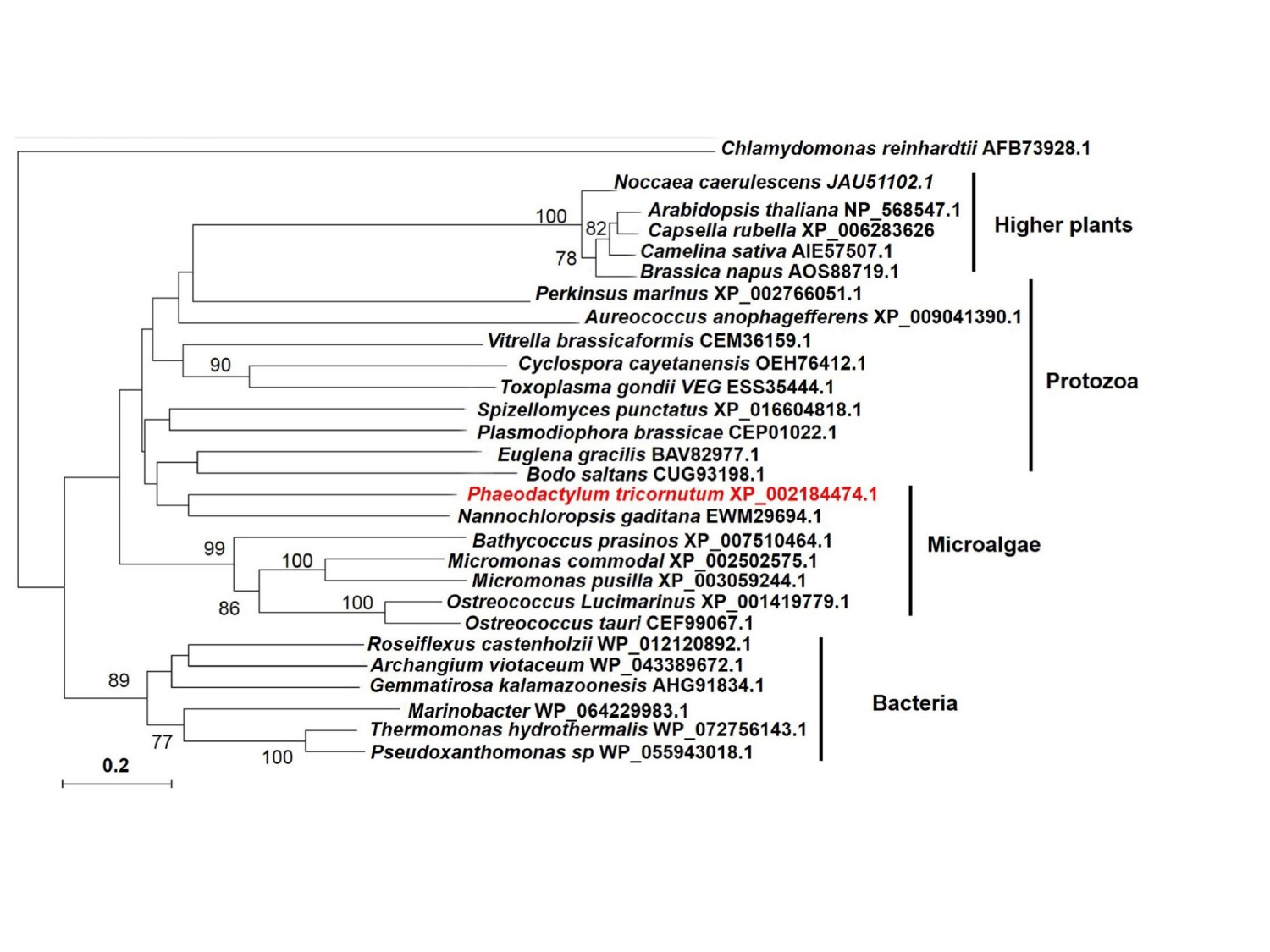

## Slide 2
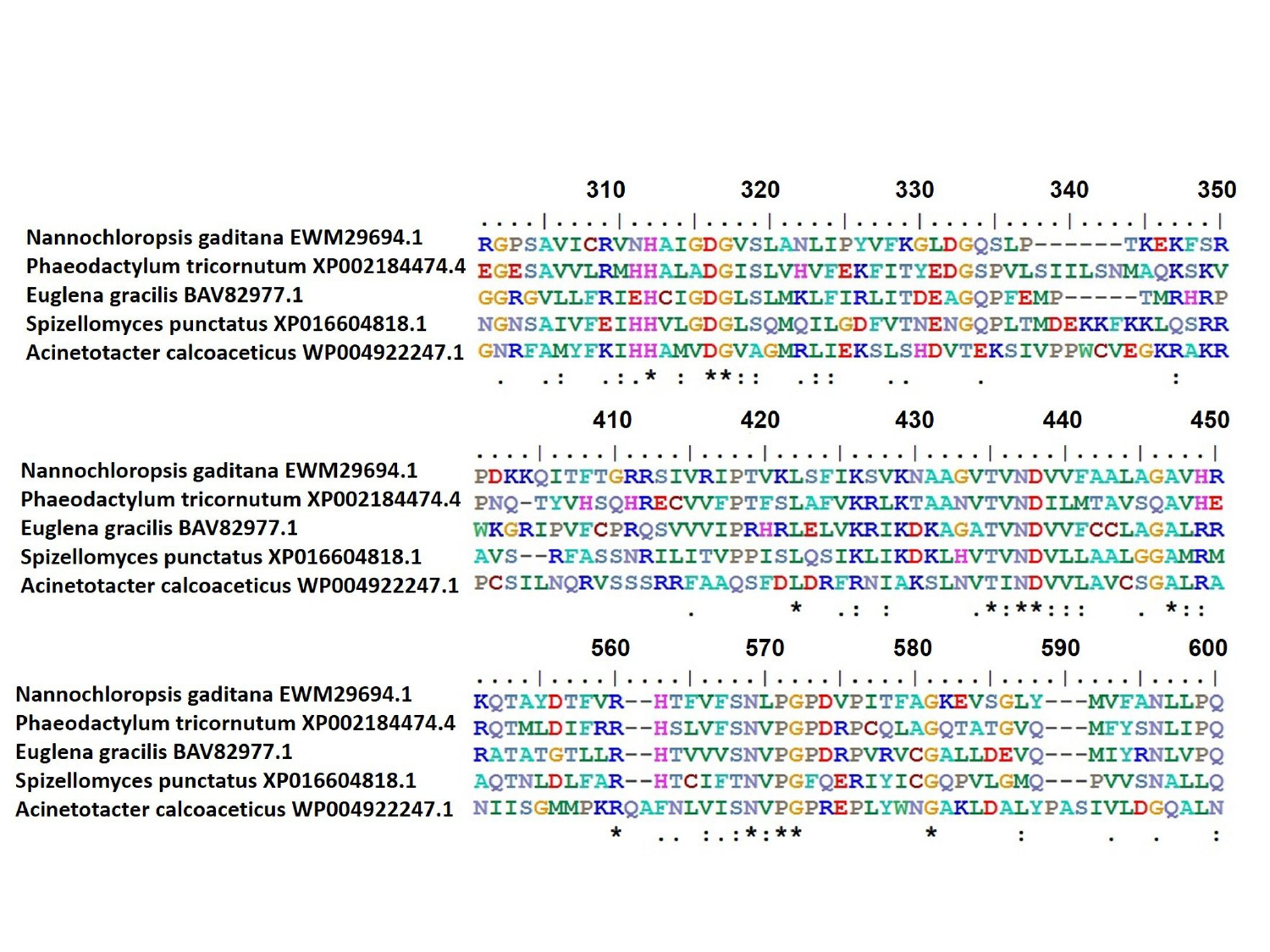

## Slide 3
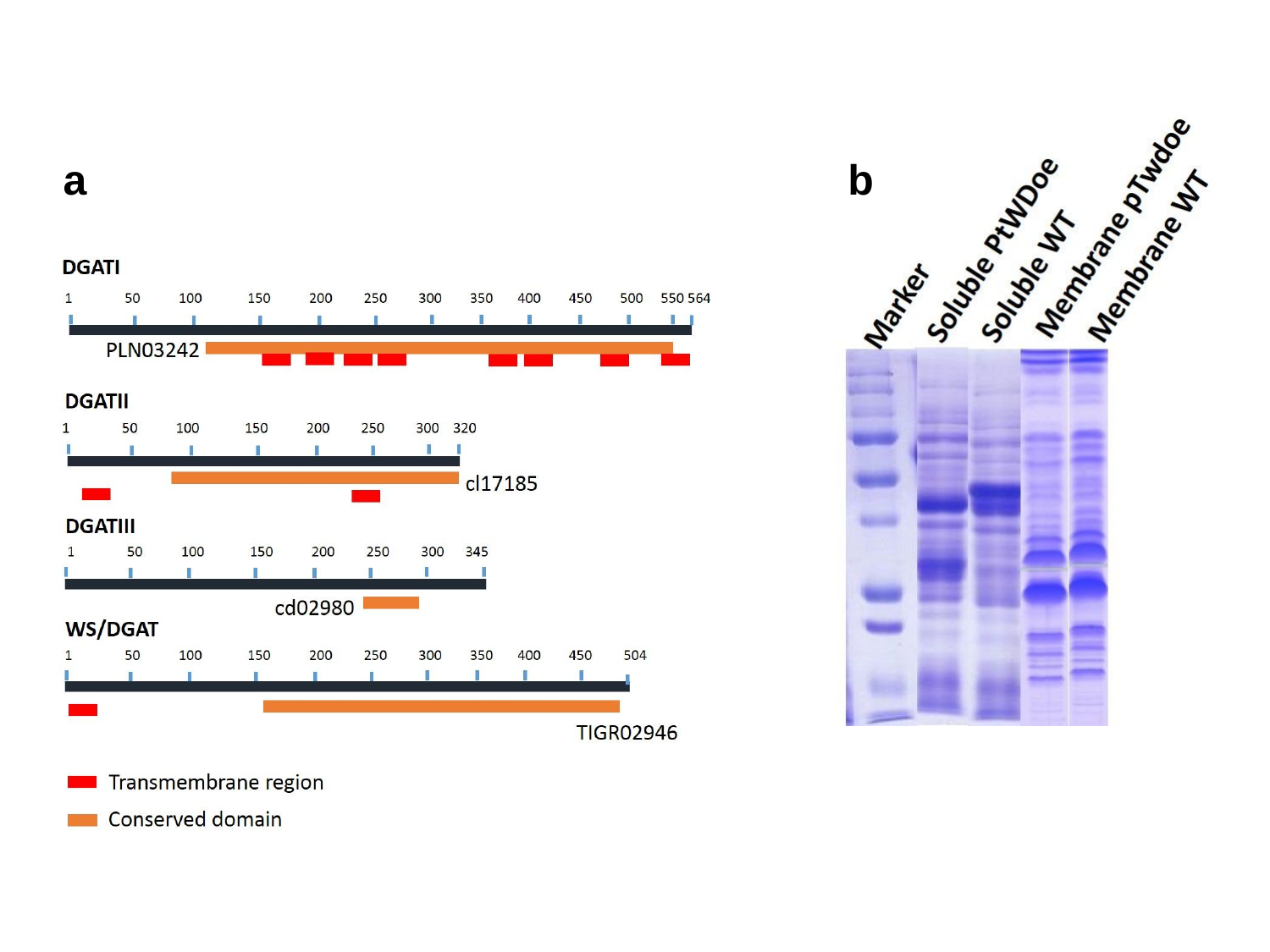

a
b

## Slide 4
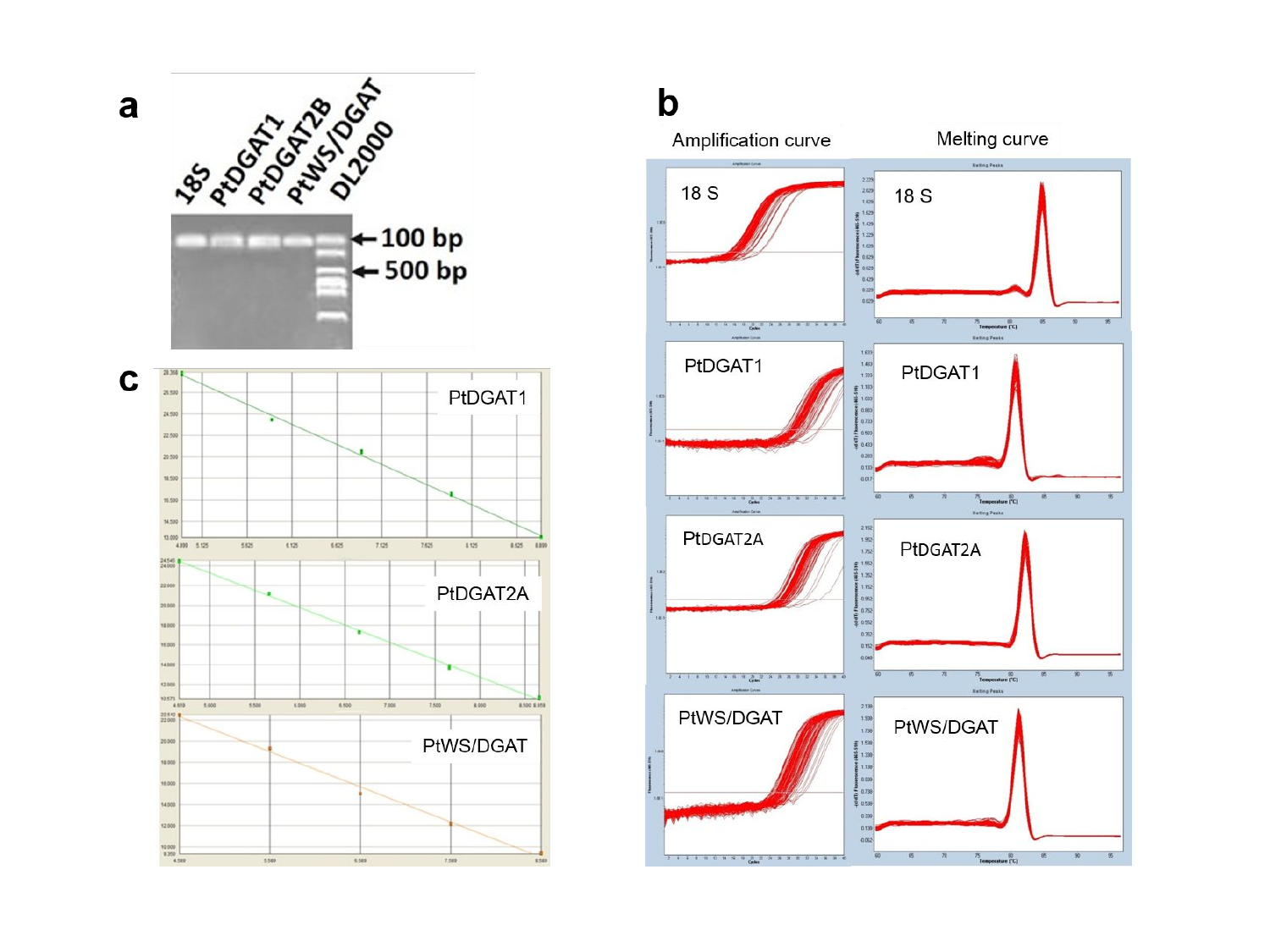

## Slide 5
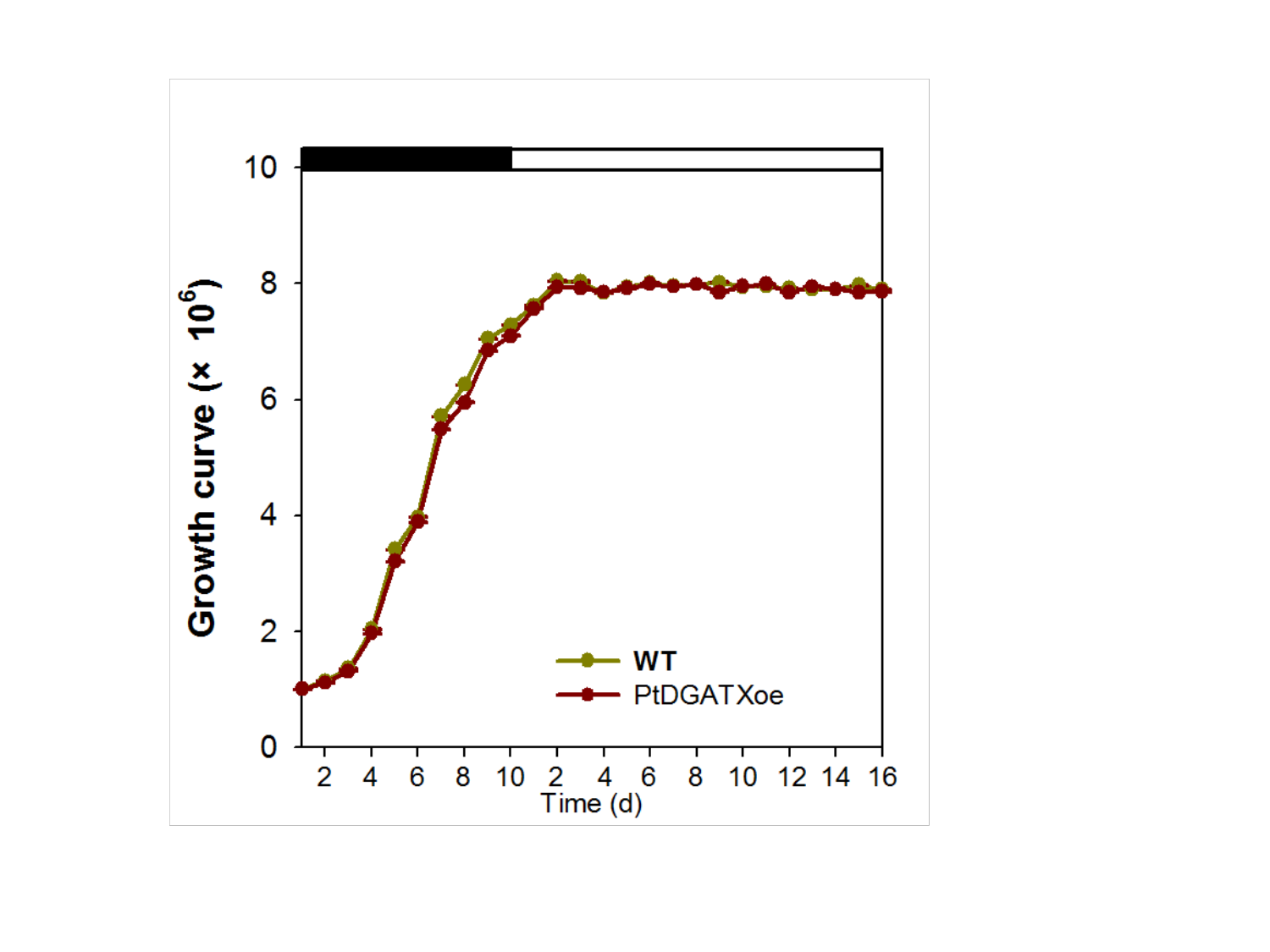

## Slide 6
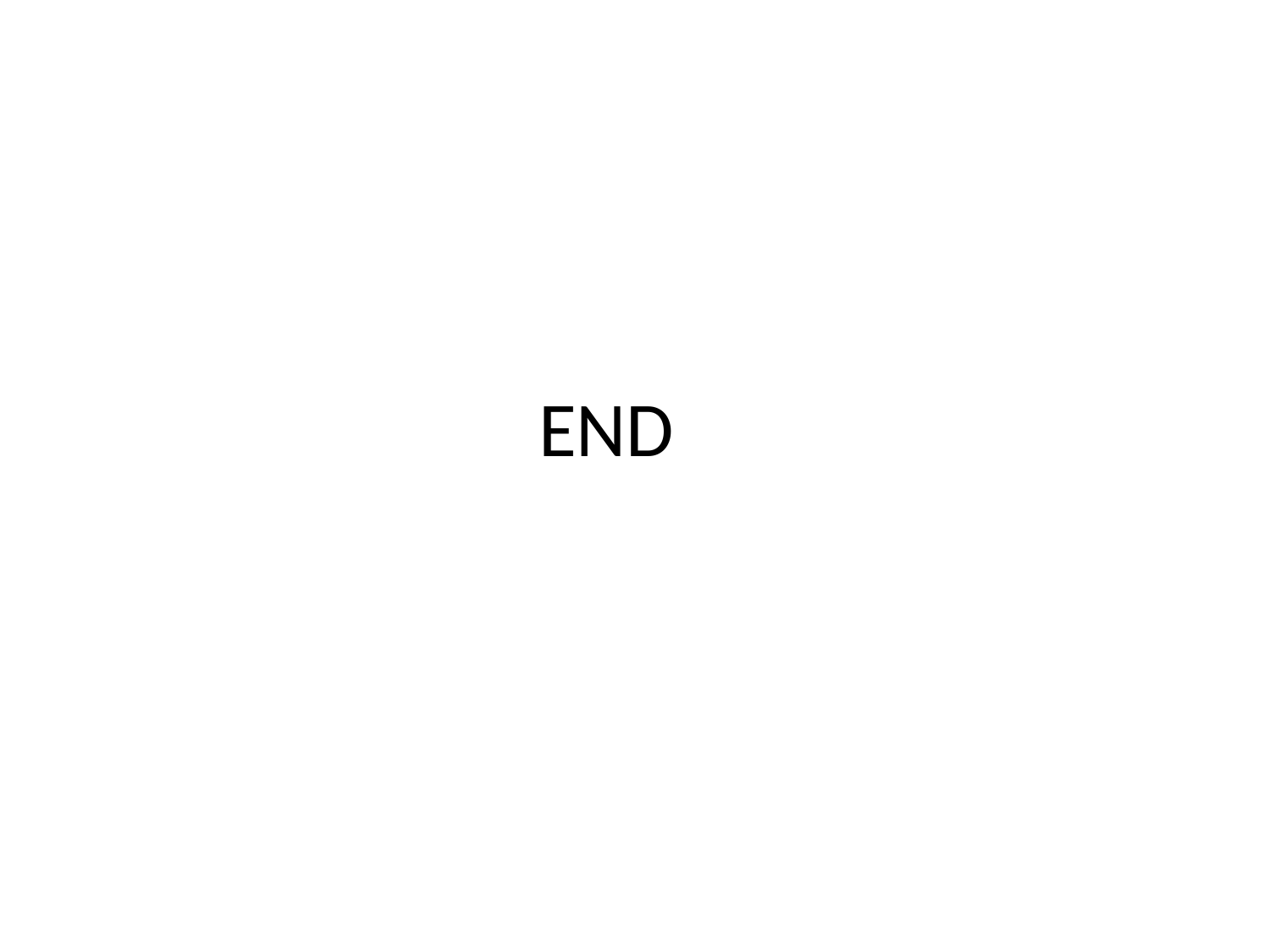

# END

Supplement: Supplementary file 2 — Additional file 2: Figure S1. Phylogenetic analysis of DGATXs. Figure S2. Multiple protein-sequence alignments of DGATXs. Figure S3. Representative topology of DGAT1s, DGAT2s, DGAT3s and WS/DGAT. Figure S4. Assessment of primers of PtDGAT1, DGAT2A, and PtWS/DGAT genes for qPCR. (a) Gel electrophoresis of the PCR product using PtDGAT1, DGAT2A and PtWS/DGAT primers. (b) Amplification curves and melting curves of PtDGAT1, DGAT2A and PtWS/DGAT primers. (c) Standard curve for absolute qPCR using vectors pMD19T-PtDGAT1, pMD19T-PtDGAT2A and pMD19T-PtWS/DGAT as templates. The vector concentrations were determined by Nanodrop ND2000 and diluted to 10−1, 10−2, 10−3, 10−4, and 10−5 for standard curve. R2 with values above 0.99 and amplification efficiencies with values above 90% were obtained for PtDGAT1, DGAT2A and WS/DGAT genes. Figure S5. Growth of WT and the PtWS/DGAToe lines under nitrogen repletion conditions (black bar) followed by nitrogen starvation (white bar). [file 13068_2018_1029_MOESM2_ESM.pptx]
