# Supplementary material for: Characterization and engineering of a dual-function diacylglycerol acyltransferase in the oleaginous marine diatom Phaeodactylum tricornutum
Source: Biotechnol Biofuels. 2018 Feb 9;11:32. doi: 10.1186/s13068-018-1029-8 (PMC5806285; doi:10.1186/s13068-018-1029-8)
Supplement: Supplementary file 3 — Additional file 3: Table S1. Primers used in this study. Table S2. The Fv/Fm of WT and PtWS/DGAToe P. tricornutum lines following nitrogen deprivation for fourteen days. [file 13068_2018_1029_MOESM3_ESM.docx]

**Table S1 Primers used in this study**

| primers | Sequences (5’-3’) |
| --- | --- |
| PtDGAT-YES2-for | AAGCTTAGGATGGATGTCTTTGGC |
| PtDGAT-YES2-rev | GGATCCTTAGAAGTACGACTGTAA |
| PtDGAT-ET28-for | GGCAGCCATATGGATGTCTTTGGCAG |
| PtDGAT-ET28-for | GTGCTCGAGTTAGAAGTACGACTGTA |
| PtDGAT-Pfcp-for | CGGGATCCATGGATGTCTTTG |
| PtDGAT-Pfcp-rev | CCCAAGCTTTTAGAAGTACGAC |
| PtDGAT-test-for | ATGGATGTCTTTGGCAGC |
| PtDGAT-test-for | TTAGAAGTACGACTGTAAATT |
| *bar*-test-for | ATGAGCCCAGAACGACGC |
| *bar*-test-rev | TCATCAAATCTCGGTGACG |
| *bar*-probe-for | GCACCATCGTCAACCACTA |
| *bar*-probe-rev | CAGAAACCCACGTCATGC |
| PtDGAT3- probe -for | ACCTGCAAGAGTCGCTCTCGAC |
| PtDGAT3- probe -rev | ACGTGAGGACCTTGGTAGCAT |
| PtDGAT1-RT-for | TTATGCACGAGGTGCTTG |
| PtDGAT1-RT-rev | CCGGGAATTTGCGATAGAG |
| PtDGAT2B-RT-for | GACTCATCTCCCCGCTCAT |
| PtDGAT2B-RT-rev | AAACGACAGTGCTCCATGC |
| PtDGAT3-RT-for | AGCTCCCACAACAATCATC |
| PtDGAT3-RT-rev | CGTGAAAGCAAGCATAGGT |

**Table S2. The *Fv*/*Fm* of WT and PtWS/DGAToe *P. tricornutum* lines following nitrogen deprivation for fourteen days.**

| Days | Wild type | PtWS/DGAToe |
| --- | --- | --- |
| 0 | 0.625±0.137 | 0.624±0.124 |
| 1 | 0.637±0.115 | 0.641±0.142 |
| 2 | 0.614±0.213 | 0.622±0.041 |
| 3 | 0.483±0.034 | 0.497±0.108 |
| 4 | 0.481±0.121 | 0.478±0.086 |
| 5 | 0.479±0.095 | 0.479±0.012 |
| 6 | 0.457±0.184 | 0.461±0.139 |
| 7 | 0.413±0.073 | 0.423±0.145 |
| 8 | 0.405±0.057 | 0.411±0.157 |
| 9 | 0.391±0.094 | 0.389±0.025 |
| 10 | 0.376±0.142 | 0.381±0.014 |
| 11 | 0.311±0.138 | 0.314±0.045 |
| 12 | 0.324±0.087 | 0.318±0.028 |
| 13 | 0.323±0.031 | 0.316±0.037 |
| 14 | 0.317±0.021 | 0.312±0.065 |
